# Besnoitiosis in donkeys: an emerging parasitic disease of equids in Italy

**DOI:** 10.1007/s00436-021-07089-9

**Published:** 2021-03-16

**Authors:** Luca Villa, Alessia Libera Gazzonis, Carlos Diezma-Diaz, Chiara Perlotti, Sergio Aurelio Zanzani, Francesco Ferrucci, Gema Álvarez-García, Maria Teresa Manfredi

**Affiliations:** 1 Department of Veterinary Medicine, Università degli Studi di Milano, Via dell’Università 6, 26900 Lodi, Italy; 2 SALUVET, Animal Health Department, Faculty of Veterinary Sciences, Complutense University of Madrid, Ciudad Universitaria s/n, 28040 Madrid, Spain; 3 Brescia, Italy; 4 Department of Health, Animal Science and Food Safety, Università degli Studi di Milano, Via dell’Università 6, 26900 Lodi, Italy

**Keywords:** *Besnoitia* spp., Donkey, Case report, Clinical features, Serology, PCR

## Abstract

Besnoitiosis is an emerging parasitic disease of equids. Italy is one of the few European countries where the circulation of *Besnoitia* spp. antibodies was demonstrated. In this study, a case of clinical besnoitiosis in two donkeys in northern Italy is reported. The two animals were clinically examined. Serum and blood samples were analyzed for the detection of *Besnoitia* spp. antibodies and for hematology, biochemistry, and enzyme activity, respectively. ITS-1 PCR and sequencing were carried out on DNA extracted from skin biopsies. Clinical examination revealed numerous scleral pearls in eyes of both animals; alopecia and hyperkeratosis with skin nodules in the region of the neck, hind leg, and on the pinnae were detected. No cysts were evidenced by endoscopy in respiratory and genital tracts. Both animals resulted seropositive to *Besnoitia* spp. antibodies by Western Blot. Hematology evidenced light anemia, leukocytosis with eosinophilia, and lymphocytosis; biochemistry and enzyme activity revealed hypoalbuminemia with decreased albumin/globulin ratio and elevated alkaline phosphatase values. Parasitic DNA extracted from skin biopsies of both donkeys demonstrated a homology of 100% with *Besnoitia* spp. This first clinical case of besnoitiosis in two donkeys in Italy both confirms the circulation of *Besnoitia* spp. in Italian equids and demonstrates that the distribution area of equine besnoitiosis in Europe could be wider than expected. Further studies are needed to infer its relevance, in relation to seroprevalence and clinical disease, and to identify the species of *Besnoitia* infecting donkeys. Besnoitiosis may be a neglected disease of donkeys in Europe: an early and accurate diagnosis is fundamental to implement adequate control measures to prevent a “silent” spread of *Besnoitia* spp. infection in equids populations.

**Supplementary Information:**

The online version contains supplementary material available at 10.1007/s00436-021-07089-9.

## Introduction

Among 10 recognized species, the genus *Besnoitia* includes four closely related species (*B. besnoiti*, *B. caprae*, *B. bennetti*, and *B. tarandi*) infecting domestic and wild ungulates (cattle, goats, equids, and deers, respectively). *Besnoitia besnoiti*, the most reported species in Europe, is the causative agent of bovine besnoitiosis. The disease is chronic and debilitating, characterized by both cutaneous and systemic manifestations, compromising animal welfare, and responsible for economic losses on affected farms (Alvarez-Garcia et al. 2013; Cortes et al. 2014). Bovine besnoitiosis is a (re)emerging disease of cattle in Europe, with an increasing of both the geographical distribution and the number of cases of infection (EFSA 2010).

Besnoitiosis in equids is caused by *Besnoitia bennetti*. The life cycle of the parasite is not completely clear: in fact, the definitive host and the mode of transmission in equine infection remain still unknown (Dubey et al. 2005). Clinical signs and lesions are similar to those observed in bovine besnoitiosis. Indeed, the disease is characterized by multifocal white pinpoint miliary parasitic cysts in the skin of the face and body, in the nares, on the pinnae, and on the limbs and perineum. *Besnoitia* lesions are frequently found on mucous membranes, particularly in the upper respiratory tract. A typical feature of the disease is the development of parasitic cysts within the sclera and conjunctiva of the eye (scleral pearls). With the progression of the disease, the infected animals develop poor hair coat and skin lesions consisting of alopecia, hypotrichosis, hyperpigmentation, thickening, and crusting, involving the face, muzzle, eyes, ears, neck, flanks, legs, and perineum. Some infected donkeys remain otherwise healthy, others become cachectic and debilitated (Dubey et al. 2005; Ness et al. 2012).

The disease was historically limited to donkeys and horses in Africa, where outbreaks of the disease were reported in both species (Bennett 1927; Schulz and Thorburn 1955; Bigalke 1970; Van Heerden et al. 1993). Outside of Africa, outbreaks of *Besnoitia* spp. infection were reported in donkeys in the USA where besnoitiosis may be considered an emerging disease of these equids (Terrell and Stookey 1973; Davis et al. 1997; Dubey et al. 2005; Elsheikha et al. 2005; Ness et al. 2012; Ness et al. 2014). Concerning Europe, the first case of besnoitiosis in a horse was reported in northern France (Henry and Masson 1922). Recently, the disease was suspected in seven donkeys from southern Spain since tissue cysts were detected by histopathology (Zafra et al. 2013). Clinical cases of besnoitiosis were also reported in two and 20 donkeys in Belgium and the UK, respectively (Liénard et al. 2018; Elsheikha et al. 2020): in both these reports, the diagnosis was molecularly confirmed. Furthermore, *Besnoitia* spp. specific antibodies were detected in equids in Spain (Gutierrez-Exposito et al. 2017), Portugal (Waap et al. 2020), and also in Italy (Villa et al. 2018) where outbreaks of bovine besnoitiosis were previously reported (Gentile et al. 2012; Gazzonis et al. 2014, 2017; Villa et al. 2019, 2020).

This study reports the diagnosis of a case of besnoitiosis in two donkeys, for the first time in Italy, using clinical, serological, and molecular tools.

## Materials and methods

### Background

In March 2019, a private veterinarian (C.P.) referred to the Parasitology Laboratory (Department of Veterinary Medicine, University of Milan, Lodi, Italy) two donkeys with poor body condition and suspected skin lesions. The animals, two 1-year-old Amiatina donkeys, one male and one female, were reared by a private owner as companion animals in Brescia suburbs (northern Italy) (45° 29′ 48″ N 10° 12′ 18″ E) after being purchased from a farm located in the mountains nearby (Val Camonica, Brescia, Italy) (46° 00′ 27″ N 10° 20′ 51″ E) 3 months before. The two donkeys were kept in a fenced area during the day and recovered indoor during the night.

### Clinical examination and sample collection

The two donkeys were hospitalized in the facilities of the Equine Isolation Unit of the Veterinary Teaching Hospital of the University of Milan (Lodi, Italy). Here, the animals were clinically examined, and body temperature (°C) was measured. The presence of tissue cysts ascribable to besnoitiosis was checked in the skin, sclera, and the vulva for the female donkey. The coat and skin of the animals were inspected for the presence of ectoparasites (lice and mites), eventually identified according to morphological characteristics (Taylor et al. 2015). Endoscopy of the upper respiratory tract and vagina for the female animal was performed using an equine flexible video-endoscope (Fujinon - DBE EN-450P5/20, Fujifilm, Balcatta, Australia). Bronchoalveolar lavage was also carried out. Each donkey was injected with Detomidine HCl (Medesan, Virbac S.r.l., Milan, Italy; 0.2 mg/kg b.w. intravenous) used as a sedative.

Blood samples were collected both in tubes with EDTA and without anticoagulants by puncturing of the jugular vein using a Vacutainer® sterile collection system. Once in the laboratory, sera were separated by centrifugation (2120*g*, 15 min) and then stored at − 20 °C until serological analysis. Skin biopsies were collected by punch biopsy from the region of the neck and hindlimb using a 6-mm disposable punch instrument (GIMA S.p.A., Gessate, Italy). Tissue samples were mechanically homogenized and stored at − 20 °C for subsequent molecular analyses. Fecal samples were collected individually from both donkeys, stored refrigerated at + 4 °C, and analyzed within 24 h.

After hospital discharge, the two donkeys were regularly revisited for clinical follow-up.

### Serology

Western Blot for *Besnoitia* spp. was performed and interpreted as previously described (Villa et al. 2018). Briefly, a total of 4 × 10^7^ *B. besnoiti* tachyzoites under non-reducing conditions were employed for electrophoresis. Tachyzoite antigens were transferred to nitrocellulose membranes and incubated with sera from the two donkeys at a 1:20 dilution, followed by a peroxidase-conjugated protein G diluted at 1:100 (Sigma-Aldrich®, Saint Louis, USA). Positive and negative control sera, both from donkey (Villa et al. 2018) and bovine (Villa et al. 2019), were included. The presence of at least three bands in at least two of the three principal antigenic areas (area I: 72.5, 58.9, and 51.4 kDa; area II: 38.7, 31.8, and 28.5 kDa; area III: 23.6, 19.1, 17.4, 14.5 kDa) was considered a positive result for *Besnoitia* spp. infection (Garcia-Lunar et al. 2013).

Immunofluorescence antibody tests for other protozoal diseases, including *Babesia caballi*, *Theileria equi*, *Toxoplasma gondii*, *Neospora* spp., and *Leishmania infantum* were also carried out (MegaFLUO®, MEGACOR Diagnostik GmbH, Lindau, Germany), following the manufacturer’s instructions. Cut-off values suggested by the producer were used for all pathogens, except for *L. infantum*, for which a cut-off of 1:40 was applied.

### Molecular analysis

Tissue sample homogenates were processed to extract genomic DNA using a commercial kit (NucleoSpin® Tissue, Macherey-Nagel, Berlin, Germany), following the manufacturer’s instructions. DNA samples were analyzed using a conventional PCR targeting a region of 231 bp of the ITS-1 region as described by Cortes et al. (2007). Positive and negative (non-template) controls were inserted in each run: positive samples consisted of DNA extracted from skin biopsies’ samples from a bovine besnoitiosis chronically affected dairy cow (Villa et al. 2019). PCR products were run on 1.5% agarose gel containing 0.05% ethidium bromide in TBE buffer electrophoresis and visualized under UV light on a transilluminator. Bands of the expected size were excised from agarose gel, purified with a commercial kit (NucleoSpin® Gel and PCR Clean-up, Macherey-Nagel, Berlin, Germany) following the manufacturer’s instructions, and finally sent for sequencing in both directions to a commercial service (Eurofins Genomics GmbH, Ebersberg, Germany). Obtained sequences were manually assembled and compared to available *Besnoitia* spp. sequences using BLASTn software (https://www.ncbi.nlm.nih.gov/blast/).

### Hematology, biochemistry, and enzyme activity

On blood samples preserved in tubes with EDTA, hematological analyses were performed within 24 h from the collection time, using the automated laser hematology analyzer ADVIA 120 with multispecies software for veterinary use (Siemens Healthcare Diagnostics, Milan, Italy). The following hematological parameters were included: red blood cells (RBC), hemoglobin (Hb), hematocrit (Ht), mean corpuscular volume (MCV), mean corpuscular hemoglobin (MCH), mean corpuscular hemoglobin concentration (MCHC), white blood cells (WBC), neutrophils, monocytes, eosinophils, basophils, lymphocytes, platelet count (PLT). The leukocyte differential provided by the instrument was checked microscopically on Romanowsky stained blood smears (Dif-stain kit, Titolchimica S.p.A., Rovigo, Italy).

Biochemistry and enzyme activity analyses were carried out on serum with the automated analyzer BT3500 (Biotecnica Instruments S.p.A., Rome, Italy) using reagents, controls, and calibrators provided by Futurlab S.r.l. (Limena, Italy). The following analytes were measured (acronyms [asneeded] followed by methods in parenthesis): albumin (bromochresol green), alkaline phosphatase (ALP, kinetic IFCC), aspartate aminotransferase (AST, kinetic IFCC), creatinine (Jaffè), creatine kinase (CK, kinetic IFCC), glucose (GOD-POD), total proteins (modified biuret), urea (urease). Globulin concentration was calculated by subtracting albumin from total proteins whereas the albumin:globulin ratio (A/G) by dividing albumin by globulin.

### Quantitative copromicroscopic examination

Quantitative coprological examination was performed using FLOTAC Dual Technique. Flotation solutions of satured sodium chloride (specific gravity 1200) and zinc sulfate (specific gravity 1.350), recommended for the detection of nematodes, cestodes, and trematodes eggs, nematodes larvae, and coccidian oocysts, were used (Cringoli et al. 2010).

## Results

**Fig. 1:**
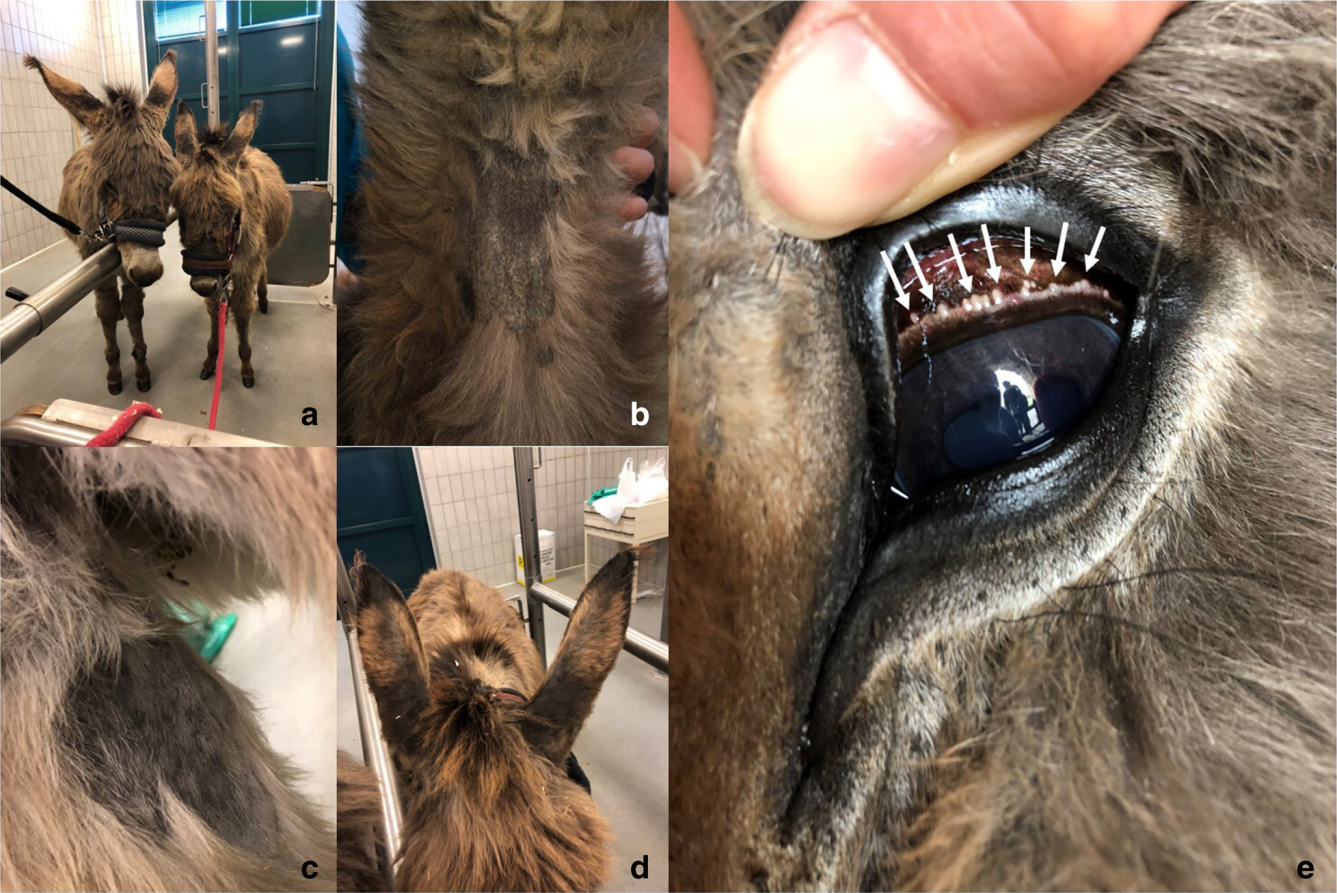
**a** Two donkeys affected by besnoitiosis showed poor body condition and presented dull and rough haircoat. **b** Alopecia and hyperkeratosis on the neck of the female donkey. **c** Alopecia and hyperkeratosis on the hind leg of the male donkey. **d** Alopecia on the pinnae of the female donkey. **e** Numerous typical scleral pearls (indicated by the arrows) in the eye of the male donkey

The two donkeys referred for suspected besnoitiosis were in poor body condition and presented dull and rough haircoat (Fig. 1.a). Alopecia and hyperkeratosis with skin nodules in the region of the neck (Fig. 1.b), hind leg (Fig. 1.c), and on the pinnae (Fig. 1.d) were detected. Skin nodules were solid, consistent, non-fistulizing, and with a size of about 0.5–1 cm. The surrounding skin was clear with no signs of reactive. A few specimens of blood-sucking lice, morphologically identified as *Haemotopinus asini*, were present on the coat of both donkeys. No lesions due to rubbing or scratching were found. Numerous typical scleral pearls in the eyes of both animals were detected (Fig. 1.e). No cysts were revealed by endoscopy in the nares and the mucosa of larynx and nasopharynx. In the female donkey, no cysts were detected in the vulva and vagina neither by visual inspection nor by endoscopy. Body temperature was normal in both donkeys (37.3 and 37.5 °C in male and female animals, respectively).

**Fig. 2:**
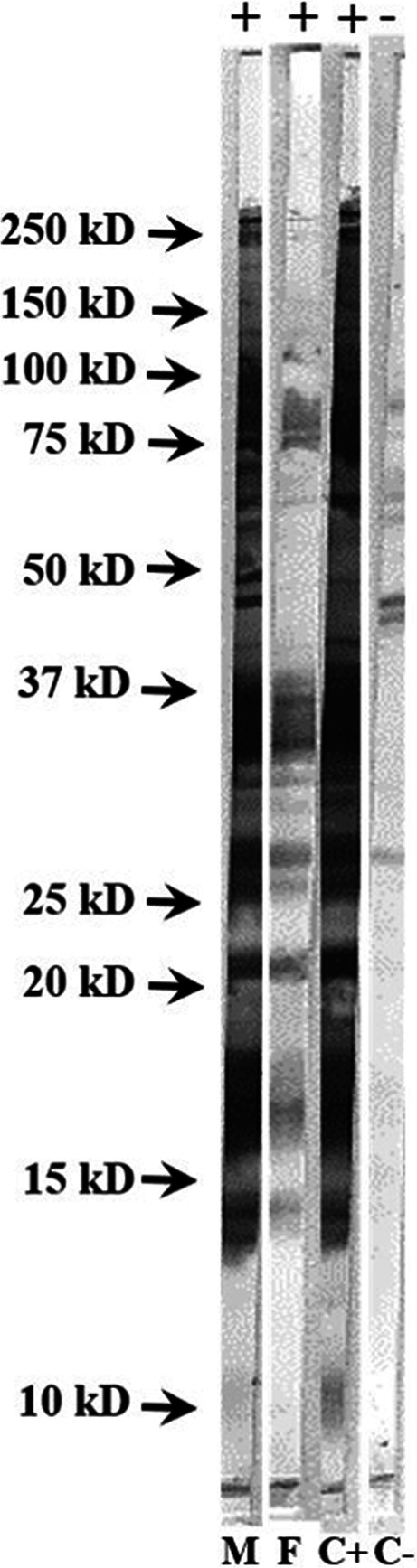
Pattern of recognition of *Besnoitia* spp. tachyzoite antigens in serum samples from donkeys affected by besnoitiosis by Western Blot. M male donkey, F female donkey, C+ positive control, C- negative control

Both animals resulted seropositive to *Besnoitia* spp. according to Western Blot results (Fig. 2).

Skin biopsies collected from both donkeys resulted positive for the presence of parasitic DNA. Sequencing of 231-bp PCR-fragments demonstrated no nucleotide variations between the two sequences (100% identity), and homology of 100% with *Besnoitia* spp. sequences deposited in GenBank. In particular, ITS-1 sequencing revealed that the T insertion at position 148, reported for *B. bennetti* (Liénard et al. 2018), was not evidenced, suggesting that our isolates may be identified as another *Besnoitia* species, i.e., *B. besnoiti*, *B. caprae*, or *B. tarandi*, even if in any case *B. bennetti* could not be discarded (Supplementary File 1). However, a conclusive species identification could not be achieved. The obtained sequence was submitted to GenBank under accession number MW520183. Therefore, based on both the clinical examination and the results obtained by serology and molecular analysis, the diagnosis of besnoitiosis was confirmed in both donkeys.

Regarding the remaining laboratory tests, the animals were seronegative to *B. caballi*, *T. equi*, *T. gondii*, *Neospora* spp., and *L. infantum*. Hematology revealed light anemia in the male donkey, leukocytosis with eosinophilia, and lymphocytosis in both animals. Biochemistry and enzyme activities evidenced hypoalbuminemia with decreased A/G ratio and elevated ALP values in both donkeys (Table 1). Finally, copromicroscopic analyses revealed that both donkeys were highly infected by strongyles (EPG = 1716 and 916 in male and female donkey, respectively), *Parascaris equorum* (EPG = 244 and 1012 in male and female donkey, respectively), and *Dictyocaulus arnfieldi* (LPG = 16 and 60 in male and female donkey, respectively). Larvae of lungworms were also found in bronchoalveolar lavage. Then, both donkeys were dosed with ivermectin at 200 μg/kg b.w (EQVALAN® Ivermectine Paste, Boehringer Ingelheim Animal Health Italia S.p.A., Milan, Italy) and their body condition quickly improved.

**Table 1:** Results of hematological, biochemical, and enzyme activities analyses of the donkeys

	Parameter	Male	Female	Unit	Range
Hematology	RBC	**5.11**	7.15	×10^6^/μL	6.8–9.3
	WBC	**13.19**	**15.96**	×10^3^/μL	5.5–9
	Hb	**9.7**	13.5	gr/dl	11.3–16.5
	Ht	32.8	42.4	%	32–45
	MCV	**64.2**	**59.3**	μ^3^	37–59
	MCH	19.0	18.9	pg	15–19
	MCHC	**29.6**	31.8	g/dl	31–40
	PLT	140	146	×10^3^/μL	90–200
	Neutrophils	35	30	%	30–65
	Lymphocytes	**51**	**50**	%	25–40
	Monocytes	2	2	%	1–3
	Eosinophils	**12**	**18**	%	0–2
	Basophils	0	0	%	0–1
	Neutrophils	4.6	4.8	×10^3^/μL	2.2–8.1
	Lymphocytes	**6.7**	**8**	×10^3^/μL	1.7–5.8
	Monocytes	0.3	0.3	×10^3^/μL	0–1
	Eosinophils	**1.6**	**2.9**	×10^3^/μL	0–0.8
	Basophils	0	0	×10^3^/μL	0–0.3
Biochemistry and enzyme activity	Urea	41	37	mg/dL	15–45
	Creatinine	0.7	0.7	mg/dL	<1.6
	Glucose	38	54	mg/dL	80–110
	Total protein	5.5	6.9	g/dL	5.5–8
	Albumin	**1.5**	**2.1**	g/dL	2.9–3.6
	Globulin	4	4.8	g/dL	2.6–4.4
	A/G ratio	**0.4**	**0.4**		0.7–1.5
	AST	122	186	U/L	<300
	ALP	**422**	**397**	U/L	<180
	CK	123	134	U/L	<180

Values in bold indicate altered parameters with respect to the reference range

After hospital discharge, the donkeys were revisited four times, 1 month apart and regularly every 6 months. Their body condition was improved; however, seropositivity and all clinical signs of besnoitiosis were still present.

## Discussion

In this study, the diagnosis of besnoitiosis in two donkeys was confirmed for the first time in Italy. The diagnosis was achieved by a multidisciplinary approach, based on clinical features and laboratory findings, including serological and molecular analyses. Moreover, blood parameters (hematology, biochemistry, and enzyme activity) and coprological examination were also performed.

The two infected donkeys were 1 year old. According to previous studies, clinical cases were reported both in young donkeys with an age range between 1 and 7 years old (Ness et al. 2012), but the disease was also reported in older animals (Dubey et al. 2005; Liénard et al. 2018; Elsheikha et al. 2020). The animals showed clinical signs typical of the disease, i.e., scleral pearls in the eyes and tissue cysts in the skin, particularly in the region of the neck and the leg and on the pinnae. As previously demonstrated, the sclera and the nares are the most common localization sites for *Besnoitia* spp. lesions in donkeys (Elsheikha et al. 2005, Ness et al. 2014). However, no lesions in the nares were detected. Endoscopy did not reveal lesions in the larynx and nasopharynx; indeed, these lesions were identified only in half of confirmed case reports (Ness et al. 2012). Furthermore, parasitic cysts were not detected in the vulvar mucous membranes, while these signs were described in previous studies (Dubey et al. 2005; Ness et al. 2012). The presence of skin lesions is widely reported in donkeys affected by besnoitiosis, even if the disease severity ranges from mild signs with animals in good condition to more serious clinical forms also leading to compromised health status (Dubey et al. 2005; Elsheikha et al. 2005; Ness et al. 2012, 2014). Only mild clinical signs were evidenced in these animals, probably due to parasite dose of infection and host immunity. The donkeys should be followed up over time to discern the evolution of the disease.

Both animals resulted seropositive to *Besnoitia* spp. antibodies by Western Blot, whereas they were seronegative to other protozoal diseases (*B. caballi*, *T. equi*, *T. gondii*, *Neospora* spp., and *L. infantum*). Molecular biology confirmed the presence of parasitic DNA in skin biopsies collected from both donkeys and sequencing demonstrated the identity of *Besnoitia* spp.

The donkeys showed some hematological disorders as leukocytosis with eosinophilia and lymphocytosis; besides, the male donkey presented also light anemia. These alterations were previously reported in cases of besnoitiosis in donkeys (Dubey et al. 2005; Liénard et al. 2018). Interestingly, similar alterations in hematological parameters were reported in *B. besnoiti*–infected cattle (Langenmayer et al. 2015; Villa et al. 2021). Besides, the animals showed hypoalbuminemia, probably due to intestinal protein loss, but also inflammation since albumin acts as a negative acute-phase protein. The same alteration was reported in a donkey with besnoitiosis (Dubey et al. 2005), and in clinically affected cows (Villa et al.  2021). Both donkeys also showed elevated ALP values: this finding may be related to liver and gut suffering. Blood parameters may be useful to aid veterinarians in the diagnosis of the disease in donkeys. However, it should be considered that donkeys were infected by gastrointestinal strongyles, ascarids, and lungworms, and an infestation by blood-sucking lice was evidenced. Indeed, these parasites could influence both the blood parameters and body condition. Donkeys showed a moderate infection by *D. arnfieldi* but they did not exhibit any respiratory clinical signs; according to Matthews and Burden (2013), these equids seem to be resistant to lungworm infection in contrast to horses.

Regarding the origin of the infection, the life cycle and transmission routes are not clear for *Besnoitia* species infecting equids. In analogy to besnoitiosis in cattle, it is suspected that insects could act as mechanical vectors for the parasite and also direct contact between animals may act a role in the spread of the infection (Dubey et al. 2005; Ness et al. 2012). Particularly, the donkeys involved in the study were born in a herd living in the mountains (lower Val Camonica, Central Alps) and then moved to their current location at the age of 9 months. In the origin farm, apart from horses and donkeys, other animal species were bred, including cattle, sheep, and goats; dogs were also present. Besides, also wild species lived in the area. In this context, donkeys may have come into contact with other infected animals. Furthermore, in both locations the donkeys have been living outside during the day and recovered indoor during the night, then the contact with vector insects could have been possible also because programs for the control of insects were not applied. Finally, it should be emphasized that the current location of the donkeys is distant about 20 km from a dairy herd endemically infected with bovine besnoitiosis and it cannot be excluded that the animals acquired the infection in their current residence (Villa et al. 2019). Indeed, epidemiological data together with molecular results may suggest that the donkeys could have been infected by *B. besnoiti*, the species infecting cattle*.* Indeed, analogously, *B. besnoiti* etiology was demonstrated by genotyping in a roe deer affected by systemic besnoitiosis (Arnal et al. 2017), thus confirming its possibility to infect and cause clinical disease in species other than cattle. However, even if *B. bennetti* was not reported in Italy so far, the lack of T insertion at position 148 of ITS-1 sequencing is not sufficient to discard *B. bennetti*, because it could be just a variation of the isolate. For all these reasons, the etiological diagnosis of *B. besnoiti* in these donkeys is just a hypothesis since in this case it was not possible to achieve a conclusive species identification. Nonetheless, the possibility for donkeys to get infected with *B. besnoiti* would be of concern for the transmission of besnoitiosis among cattle and equids populations.

Besnoitiosis in equids was previously reported in Europe: recently, besnoitiosis was diagnosed in two donkeys in Belgium, and *B. bennetti* was recognized by partial rDNA sequencing (Liénard et al. 2018). In the UK, the infection of *B. bennetti* was confirmed by microsatellite genotyping of DNA isolated from a dermal mass in one out of 20 infected donkeys (Elsheikha et al. 2020). Furthermore, serosurveys for *Besnoitia* spp. infection were performed in equids from southern Europe and donkeys seem to be more affected than horses (Gutierrez-Exposito et al. 2017; Waap et al. 2020). In Italy, the circulation of *Besnoitia* spp. infection both in horses and donkeys was recently confirmed: a value of seroprevalence of 2.1% increasing up to 22.2% if considering only donkeys was detected (Villa et al. 2018).

As previously underlined (Dubey et al. 2005), parasite antibodies were found in equids without any clinical signs of the disease, suggesting that *Besnoitia* spp. infection could be more spread than realized in the USA. Similarly, also in Europe, the presence of anti-*Besnoitia* spp. specific antibodies was reported in apparently healthy horses and donkeys (Gutierrez-Exposito et al. 2017; Villa et al. 2018; Waap et al. 2020). For this reason, in analogy to bovine besnoitiosis, it should also be considered that infected animals without detectable clinical signs and macroscopic lesions, i.e., subclinically infected animals, could be more frequently found than clinically affected animals. In this way, only a small proportion of seropositive animals develop clinical signs. Instead, a larger subset includes seropositive subclinically infected animals without any clinical sign: this category poses a huge risk for parasite transmission, being a source of infection for the other animals (Villa et al. 2019).

Besnoitiosis in equids could be almost as spread as bovine besnoitiosis in Europe. However, due to difficulties in the diagnosis, besnoitiosis could be underdiagnosed and underreported, thus favoring a silent spread of the disease in European equids. Besnoitiosis should be included among differential diagnoses when detecting skin lesions in equids: indeed, ectoparasites, such as lice in this case, can be frequently found, and might preclude further investigations for *Besnoitia* spp. infection diagnosis. Besides, other frequently found parasitic infections could conceal *Besnoitia* spp. infection (e.g., parasites causing poor body condition, as ascarids or strongyles, or nematodes of the respiratory tract). A better understanding of the epidemiology of *Besnoitia* spp. infection in the donkey populations in Italy and in Europe would be advisable. It is to be discerned if this case report of clinical besnoitiosis in Italian donkeys is an unusual cluster of infection or may reflect a wider distribution of subclinical infections, largely undetected to date, since the wide-spread distribution of *Besnoitia* spp. infection in equids could be of concern for all Europe. To date, the species implicated in cases of besnoitiosis in southern Europe remains unknown: therefore, parasite isolation, characterization by microsatellite genotyping, whole-genome sequencing, and in vitro studies should be further performed to discern *Besnoitia* species’ identity. Studies are needed to infer the relevance of besnoitiosis in equids in Europe, both in relation to the seroprevalence, but also to the clinical infection, considering the need to investigate parasite biology and transmission routes.

## Conclusions

In the study, the diagnosis of besnoitiosis was achieved in two donkeys in Italy. Both animals showed typical clinical signs, anti-*Besnoitia* spp. antibodies were evidenced in both animals by Western Blot, and some alterations in hematological and biochemical parameters were detected. Molecular analysis confirmed the presence of parasitic DNA belonging to *Besnoitia* spp. from skin biopsies of both donkeys.

This first clinical case of besnoitiosis in two donkeys in Italy confirms the circulation of *Besnoitia* spp. in Italian equids. Besides, together with other recent clinical and serological studies, it is confirmed the circulation of the parasite in equids in Europe.

*Besnoitia* spp. infection may be more common in equids in Italy and in Europe than realized since it is scarcely known and then diagnosed by veterinarians, who should be aware of this parasitic disease of equids due to the consequences for health and well-being of animals. Knowledge of clinical features of besnoitiosis in horses and donkeys could assist clinicians in the diagnosis and prevention of the disease, since an early and accurate diagnosis, also considering the absence of vaccines and treatments, is fundamental to implement adequate control measures to prevent a “silent” spread of *Besnoitia* spp. infection in equids populations.

## Supplementary Information

ESM 1 (RTF 50 kb)

## Data Availability

The data that support the findings of this study are available from the corresponding author upon reasonable request.
