# Supplementary material for: Besnoitiosis in donkeys: an emerging parasitic disease of equids in Italy
Source: Parasitol Res. 2021 Mar 16;120(5):1811–9. doi: 10.1007/s00436-021-07089-9 (PMC8084774; doi:10.1007/s00436-021-07089-9)
Supplement: Supplementary file 1 — (RTF 50 kb) [file 436_2021_7089_MOESM1_ESM.rtf]

Alignement of ITS1


                               10         20         30         40         50         60         70         80         90        100                           
                      
JF314861 B. besnoiti  TGACATTTAA TAACAATCAA CCCTTGAATC CCTATTACAA CAATAAGCTT GCATCTCTCG TTTCGAGGGG TGCATTCGAG AAGTGTGCTG CCCTCTTGTT  
HM008988 B. caprae    .......... .......... .......... .......... .......... .......... .......... .......... .......... ..........  
AY665400 B. tarandi   .......... .......... .......... .......... .......... .......... .......... .......... .......... ..........  
MG652473 B. bennetti  .......... .......... .......... .......... .......... .......... .......... .......... .......... ..........  
JQ013812 B. bennetti  .......... .......... .......... .......... .......... .......... .......... .......... .......... ..........  
AY665399 B. bennetti  .......... .......... .......... .......... .......... .......... .......... .......... .......... ..........  
Donkey Italy Female   .......... .......... .......... .......... .......... .......... .......... .......... .......... ..........  
Donkey Italy Male     .......... .......... .......... .......... .......... .......... .......... .......... .......... ..........  

                              110        120        130        140        150        160        170        180        190        200                  

JF314861 B. besnoiti  GTCATTTTTG ACAACAAGAG CATCGCCTTC TTTTTTTTT- CCAACACCGT TTAACTAAAC CAACGATCTG TTGTTTAGCG GGCGGGGATC CACCTCCTCA  
HM008988 B. caprae    .......... .......... .......... .........- .......... .......... .......... .......... .......... ..........  
AY665400 B. tarandi   .......... .......... .......... .........- .......... .......... .......... .......... .......... ..........  
MG652473 B. bennetti  .......... .......... .......... .........T .......... .......... .......... .......... .......... ..........  
JQ013812 B. bennetti  .......... .......... .......... .........T .......... .......... .......... .......... .......... ..........  
AY665399 B. bennetti  .......... .......... .......... .........T .......... .......... .......... .......... .......... ..........  
Donkey Italy Female   .......... .......... .......... .........- .......... .......... .......... .......... .......... ..........  
Donkey Italy Male     .......... .......... .......... .........- .......... .......... .......... .......... .......... ..........  

                              210        220        230    
                      
JF314861 B. besnoiti  CTCTGCTATC ACGGATTGGT TAATACAAAC C 
HM008988 B. caprae    .......... .......... .......... . 
AY665400 B. tarandi   .......... .......... .......... . 
MG652473 B. bennetti  .......... .......... .......... . 
JQ013812 B. bennetti  .......... .......... .......... . 
AY665399 B. bennetti  .......... .......... .......... . 
Donkey Italy Female   .......... .......... .......... . 
Donkey Italy Male     .......... .......... .......... . 
